# Vitamin D Roles Across Developmental Stages in Pediatric Pneumonia: Linking Genetics, Microbiome, Maternal Status and Immunity

**DOI:** 10.1002/hsr2.72520

**Published:** 2026-05-11

**Authors:** Nurshad Ali

**Affiliations:** ^1^ Department of Biochemistry and Molecular Biology Shahjalal University of Science and Technology Sylhet Bangladesh

**Keywords:** immune maturation, maternal vitamin D, microbiome, pediatric pneumonia, vitamin D, vitamin D receptor

## Abstract

**Background:**

Pneumonia remains a leading cause of morbidity and mortality among children globally, especially in low‐ and middle‐income countries, where poor nutrition and limited access to healthcare increase vulnerability. Vitamin D deficiency is common among children and has emerged as a significant risk factor associated with respiratory infections. This review aims to synthesize current evidence on the role of vitamin D across developmental stages in pediatric pneumonia.

**Methods:**

A comprehensive literature search was conducted in PubMed, Scopus, Web of Science, and Google Scholar to identify relevant studies on vitamin D and pediatric pneumonia. Peer‐reviewed articles, including observational studies, randomized controlled trials, and reviews, were screened. Evidence was synthesized from immunology, genetics, maternal health, and microbiome research to provide an integrated perspective on vitamin D–mediated immune responses and clinical outcomes.

**Results:**

Mechanistically, vitamin D enhances host defense by inducing antimicrobial peptides such as cathelicidin and β‐defensins. It improves macrophage phagocytic function, modulates Toll‐like receptor signaling, and preserves airway epithelial barrier integrity. Vitamin D also modulates adaptive immunity by suppressing pro‐inflammatory Th1 and Th17 responses while promoting regulatory T‐cell activity and anti‐inflammatory cytokine production. Epidemiological studies consistently show an association between low serum 25‐hydroxyvitamin D levels and increased risk and severity of pneumonia, although results from supplementation trials remain heterogeneous. Recent evidence highlights additional modifiers of vitamin D–mediated immunity, including maternal vitamin D status, vitamin D receptor (VDR) genetic polymorphisms, early‐life immune programming, and respiratory microbiome interactions, which may explain variability in clinical outcomes across populations and developmental stages.

**Conclusion:**

Overall, this review provides a comprehensive framework linking vitamin D biology with immune system development in children and their risk of pneumonia. It emphasizes the importance of age‐specific supplementation strategies and well‐designed mechanistic and clinical studies to improve prevention and management.

## 1 Introduction

Pneumonia is the leading infectious cause of death in children under five, accounting for 14%–22% of all deaths in this age group worldwide (over 700,000 each year) with the highest rates found in South Asia and sub‐Saharan Africa [1, 2]. The burden of the disease is especially high in low‐ and middle‐income countries because of malnutrition, lack of vaccination, and limited access to healthcare [1]. In these settings, modifiable biological factors like micronutrient deficiencies and immune dysregulation are becoming more recognized as key contributors to the risk and severity of childhood pneumonia.

Vitamin D deficiency is recognized as a common and modifiable risk factor for respiratory infections, including pneumonia [3, 4]. Beyond its well‐known role in calcium and bone health, vitamin D is crucial for immune regulation, maintenance of epithelial barriers, and control of inflammation [5, 6]. Globally, deficiency is prevalent among children, with estimates indicating that nearly one‐third of the pediatric population has insufficient serum 25(OH)D levels, primarily due to lack of sunlight exposure, inadequate dietary sources, and maternal deficiency [7]. These deficits are particularly marked in LMICs, where malnutrition and infection rates overlap [8].

Emerging evidence suggests that maternal vitamin D status during pregnancy may affect early immune development and the risk of respiratory issues in her children [9, 10]. Moreover, early‐life epigenetic changes caused by maternal vitamin D levels or perinatal exposures can affect immune development, shaping the risk of respiratory infections in childhood [11]. This highlights the importance of considering the entire lifespan when looking at the risk of pneumonia in children.

The term “children” includes a diverse group with significant differences in vitamin D metabolism and immune function [9, 12]. Neonates and young infants mainly depend on their mothers' vitamin D stores. They also show immature innate and adaptive immune responses [9, 12]. In contrast, preschool and school‐aged children experience rapid immune development [12, 13]. During adolescence, changes in hormones and growth needs can further affect vitamin D metabolism and inflammatory responses [14]. Understanding these developmental differences is crucial for interpreting studies and clinical trials related to vitamin D and pneumonia.

**Figure 1:**
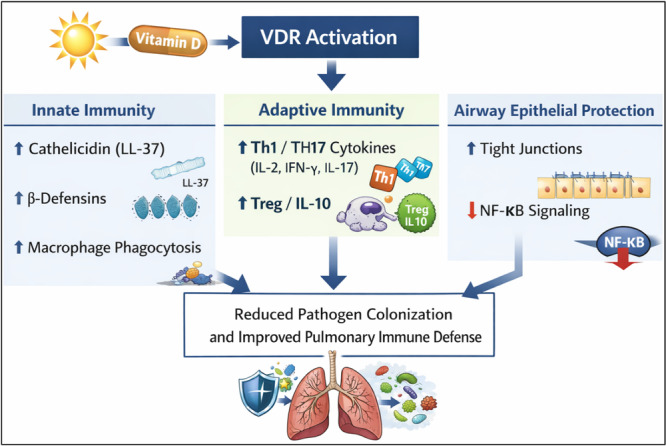
Mechanistic pathways linking vitamin D to pulmonary immune defense. Vitamin D and VDR signaling increase antimicrobial peptide production, adjust inflammatory cytokines, strengthen epithelial barrier integrity, and together lower pathogen colonization and the risk of pneumonia. VDR, vitamin D receptor; LL‐37, cathelicidin antimicrobial peptide; Th, T helper cells; Treg, regulatory T cells; IL, interleukin; IFN‐γ, interferon‐gamma; NF‐κB, nuclear factor kappa‐B.

Although many observational and interventional studies have explored this relationship, results are often variable and conflicting. Mechanistic studies suggest that vitamin D enhances both innate and adaptive immune responses by promoting the production of antimicrobial peptides (such as cathelicidin and β‐defensins), improving macrophage function, and reducing proinflammatory cytokine activity [7, 15] as illustrated in Figure 1. However, it is not fully clear how these immune mechanisms interact with the development of the immune system and other biological factors to affect the risk of pneumonia in children.

While several previous reviews have outlined the links between vitamin D deficiency and respiratory infections, few have included new biological insights that might explain the differences in outcomes. This review provides a broader framework by synthesizing evidence from various areas including pediatric immunology, genetic differences in vitamin D signaling pathways, maternal vitamin D levels and early immune development, and the interactions between vitamin D and the respiratory microbiome.

By incorporating these perspectives and looking at developmental differences from the neonatal period to adolescence, this review provides a broad overview at how vitamin D deficiency leads to immune problems and increases the risk of pneumonia in children. Specifically, the review looks at (i) the molecular and immune mechanisms of vitamin D's action, (ii) the epidemiological evidence connecting vitamin D levels with pediatric pneumonia, and (iii) practical implications for prevention and treatment strategies in different pediatric age groups.

A comprehensive literature search was conducted across PubMed, Scopus, Web of Science, and Google Scholar to identify relevant studies on vitamin D and pediatric pneumonia. Peer‐reviewed observational studies, randomized trials, and reviews were screened. Evidence was synthesized from immunology, genetics, maternal health, and microbiome research to provide an integrated perspective on vitamin D–mediated immune responses.

## 2 Vitamin D Biology and Pediatric Deficiency

Vitamin D is produced in the skin upon exposure to ultraviolet B (UVB) rays from sunlight. The precursor substance 7‐dehydrocholesterol transforms into cholecalciferol (vitamin D₃), which is then converted in the liver to 25‐hydroxyvitamin D [25(OH)D], the primary form found in circulation [16]. This metabolite undergoes further hydroxylation in the kidneys via the enzyme CYP27B1 to create 1,25‐dihydroxyvitamin D [1,25(OH)₂D], the active form that manages calcium and phosphate metabolism and influences immune system functions [16]. Furthermore, it impacts respiratory health by preserving epithelial integrity and regulating inflammation in the airways [5, 6].

Vitamin D deficiency poses a significant health issue globally, affecting approximately 30%–50% of children worldwide [17]. Recent studies highlight that vitamin D deficiency is a global issue, pointing out the need for new biosensing technologies for rapid and accurate detection of vitamin D in biological samples. Electrochemical and nanomaterial‐based biosensors, in particular, have shown high sensitivity for detecting circulating 25(OH)D₃, offering promising alternatives for point‐of‐care diagnostics [18, 19]. In areas with limited sunlight, such as certain regions of Asia, Africa, and Europe, the rates of deficiency can be considerably higher. Contributing factors include insufficient dietary intake, reduced sun exposure due to urban lifestyles or cultural norms, and maternal deficiency during pregnancy, which can adversely influence neonatal vitamin D levels [20]. In tropical countries like India, prevalence rates among children can reach between 40% and 90%, highlighting the necessity for focused public health measures [21].

### 2.1 Developmental Differences in Vitamin D Metabolism and Immune Maturation

Vitamin D metabolism and immune responses vary greatly across different pediatric age groups. Neonates depend largely on their mothers' vitamin D transfer during pregnancy and show immature innate and adaptive immune responses [22, 23]. In addition, neonatal immune function may be influenced by early‐life epigenetic programming and microbial colonization, interacting with vitamin D levels and affect susceptibility to respiratory pathogens [24, 25]. Infants and preschool children undergo rapid immune development but remain more prone to viral lower respiratory infections [26, 27]. School‐aged children build stronger adaptive immunity, while adolescents experience hormonal changes that can affect vitamin D metabolism and immune responses [17, 28]. Furthermore, differences in hormonal status, growth demands, and lifestyle‐related UVB exposure during later childhood and adolescence may affect vitamin D requirement, highlighting the importance of age‐specific supplementation strategies and clinical guidelines [29, 30, 31].

## 3 Immunomodulatory Mechanisms of Vitamin D in the Lungs

Vitamin D influences lung immunity by enhancing innate defenses, adaptive responses, and epithelial barrier function — processes that are especially vital in children due to the ongoing development of their respiratory immune system (Table 1 and Figure 1).

**Table 1:** Mechanistic roles of vitamin D in pulmonary immune regulation and host defense.

Mechanistic domain	Immunological actions of vitamin D	Key molecules/pathways	Evidence type	Strengths	Limitations	References
Innate immunity – antimicrobial defense	Induces antimicrobial peptide expression and enhances macrophage phagocytic activity	Cathelicidin (LL‐37), β‐defensins, TLR2/TLR4 signaling	Human, animal, ex vivo	Strong mechanistic evidence linking vitamin D to antimicrobial peptide induction	Limited direct clinical evidence in pediatric pneumonia	[32]
Innate immunity – cytokine modulation	Suppresses excessive inflammatory responses and modulates TLR signaling	↓ TNF‐α, IL‐6, IL‐8; inhibition of NF‐κB pathway	Human and in vitro	Demonstrates anti‐inflammatory role in immune regulation	Mostly experimental studies; clinical relevance varies across populations	[33]
Adaptive immunity – T‐cell regulation	Inhibits Th1 and Th17 responses while promoting regulatory and Th2 responses	↓ IL‐2, IFN‐γ, IL‐17; ↑ IL‐10, TGF‐β	Human, animal	Strong evidence supporting immunomodulatory effects on T‐cell differentiation	Limited pediatric‐specific clinical studies	[34, 35]
B‐cell modulation	Suppresses B‐cell proliferation and plasma‐cell differentiation	Reduced antibody (IgG) production pathways	Human in vitro	Provides insight into vitamin D effects on humoral immunity	Evidence largely restricted to in vitro studies	[36]
Epithelial barrier integrity	Enhances airway epithelial barrier function and mucosal defense	Claudin‐1, E‐cadherin, occludin, MUC1	Human airway models	Supports role in maintaining respiratory epithelial integrity	Limited in vivo pediatric data	[37, 38]
Inflammation resolution	Promotes anti‐inflammatory macrophage polarization (M2 phenotype) and reduces oxidative stress	IL‐10, arginase‐1, HO‐1	Human and animal	Demonstrates potential role in controlling excessive inflammation	Translational evidence in respiratory infections remains limited	[39]
Microbiome regulation	Influences airway and gut microbiome composition and reduces pathogen colonization	Pneumococcal carriage, microbial diversity	Human and animal	Emerging evidence linking vitamin D to host–microbiome interactions	Mechanistic pathways and causality remain unclear	[40]

Innate immunity: Active vitamin D (1,25(OH)₂D) upregulates the antimicrobial peptides (AMPs) such as cathelicidin (LL‐37) and β‐defensins in airway epithelial and myeloid cells, thereby increasing direct antimicrobial activity at the mucosal surface [41, 42]. In children, levels of circulating 25(OH)D are associated with AMP concentrations in saliva and mucosal samples, linking systemic vitamin D status to localized innate defense [43]. Additionally, vitamin D enhances the differentiation of macrophages and their phagocytic capabilities, fosters autophagy and intracellular killing processes, and upregulates complement receptor expression to aid in pathogen elimination [44]. Activation of Toll‐like receptors (TLRs) on macrophages and epithelial cells promotes the expression of VDR and CYP27B1, which allows for the local conversion of 25(OH)D into 1,25(OH)₂D and establishes an autocrine loop that amplifies AMP production and infection control [41, 45].

Adaptive immunity: Vitamin D modulates T‐cell differentiation by reducing pro‐inflammatory Th1 and Th17 cytokines (such as IL‐2, IFN‐γ, IL‐17) while promoting regulatory T cells (Tregs) and anti‐inflammatory cytokines like IL‐10 [34, 35]. This rebalancing helps decrease harmful inflammation during infections and aids in recovery — a dynamic that might mitigate the severity of pneumonia in children, where excessive inflammation can lead to complications [43].

Epithelial barrier function: Vitamin D promotes the expression of tight junction proteins and maintains mucosal integrity within the airway epithelium, which reduces permeability and bacterial/viral translocation [37]. In vitro and ex vivo studies focused on children demonstrate that vitamin D alleviates barrier dysfunction and viral replication, linking the protection of epithelial integrity to diminished susceptibility and severity of infections [38].

Although mechanistic and clinical data from pediatric populations, including randomized trials, indicate that high‐dose vitamin D can reduce recurrent pneumonia in children [46], and meta‐analyses suggest a potential role for vitamin D in preventing acute respiratory infections, more mechanistic and interventional studies specifically targeting pediatric populations are needed [43, 47].

## 4 Evidence Linking Vitamin D Deficiency to Pediatric Pneumonia

**Figure 2:**
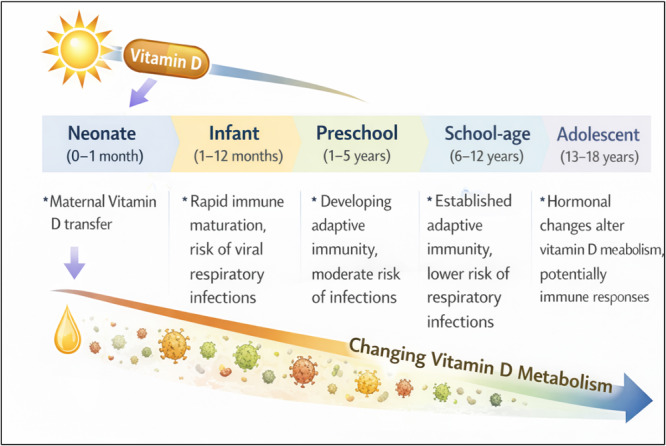
Developmental framework of vitamin D metabolism, immune maturation, and pneumonia susceptibility across pediatric age groups. This figure shows age‐related changes in vitamin D metabolism, immune development, and children's susceptibility to respiratory infections. This applies to different pediatric groups, from newborns to teenagers. It emphasizes the role of maternal transfer, immune growth, and hormonal effects on the risk of pneumonia.

A number of epidemiological and clinical studies indicate a link between vitamin D deficiency and a higher risk, occurrence, and severity of pneumonia in children (Table 2). However, the association between vitamin D levels and pneumonia risk may differ at various stages of childhood development (Figure 2). This variation may be due to differences in how vitamin D is processed, the maturity of the immune system, and exposure to respiratory germs. Numerous observational studies have shown that children with low levels of serum 25‐hydroxyvitamin D [25(OH)D] are more prone to developing pneumonia and facing prolonged illness or complications. For instance, a matched case‐control study in rural Bangladesh found that vitamin D status was linked to early childhood acute lower respiratory infections (ALRI) [50]. In Indian children, subclinical vitamin D deficiency during the first 4 months of life was identified as a significant risk factor for severe ALRI [49]. Low vitamin D levels were found to be associated with increased risk of pneumonia in children under 5 years in Nigeria [54]. A review summarizing several studies indicated that low vitamin D levels are common among most infants and children suffering from respiratory tract infections [55]. Meta‐analyses further support these findings. A combined analysis by Jolliffe et al. (2019) involving over 10,000 subjects confirmed that vitamin D deficiency significantly heightens vulnerability to ALRIs, especially in children younger than five [52].

**Table 2:** Summary of epidemiological and clinical studies evaluating vitamin D status or supplementation and pediatric pneumonia outcomes.

Study design and population	Vitamin D assessment or intervention	Outcome(s)	Main findings	Strengths	Limitations	Reference
Randomized controlled trial; *n* = 453 children (1–36 months), Kabul, Afghanistan	100,000 IU vitamin D₃ every 3 months	Recurrent pneumonia	Supplementation modestly reduced recurrence of pneumonia episodes	Well‐designed RCT conducted in high‐risk population	High bolus dosing; limited generalizability to other settings	[46]
Randomized controlled trial; *n* = 2,079 neonates, Delhi, India	Single oral dose of 50,000 IU vitamin D₃	Incidence of acute lower respiratory infection (ALRI)	No significant reduction in pneumonia or ALRI episodes	Large sample size; randomized design	Single‐dose intervention; neonatal population only	[48]
Case–control study; *n* = 150 children (2–60 months), India	Serum 25(OH)D < 50 nmol/L (deficiency)	Risk of pneumonia	Vitamin D–deficient children were more than twofold more likely to develop pneumonia	Early evidence linking deficiency with pneumonia risk	Small sample size; observational design limits causal inference	[49]
Prospective cohort; *n* = 190 children, Sylhet, Bangladesh	Serum 25(OH)D tertiles	Hospitalization for ALRI	Children in the highest vitamin D tertile had ~40% lower risk of hospitalization	Prospective design; conducted in LMIC setting	Moderate sample size; potential residual confounding	[50]
Meta‐analysis of 15 studies (0–18 years)	Serum 25(OH)D < 50 nmol/L vs ≥ 50 nmol/L	Pneumonia incidence and severity	Vitamin D deficiency associated with increased pneumonia risk	Large pooled dataset; increased statistical power	Heterogeneity across included studies	[51]
Systematic review and meta‐analysis of RCTs	Various vitamin D supplementation regimens	Acute pneumonia duration and recurrence	Mixed outcomes; benefits mainly observed in severely deficient individuals	Comprehensive evaluation of RCT evidence	Variation in dosing, populations, and study designs	[52]
Double‐blind RCT; *n* = 453 children, Afghanistan	Single high dose (100,000 IU vitamin D₃) during pneumonia episode	Recovery time and recurrence	No effect on recovery duration but reduced recurrence over 3 months	Randomized double‐blind design	Short follow‐up period; high‐dose intervention	[46]
Systematic review and meta‐analysis; LMICs, eight observational studies (*n* = 20,966)	Prevalence of vitamin D deficiency	ALRI and community‐acquired pneumonia	Vitamin D deficiency significantly associated with increased risk of pneumonia	Large pooled sample size; focus on LMIC populations	Observational studies only; heterogeneity in vitamin D assessment	[53]

Regional variations are significant. Research from low‐ and middle‐income countries (LMICs) consistently shows stronger links between vitamin D deficiency and pneumonia risk, likely due to compounding factors such as malnutrition, lack of supplementation policies, and reduced sun exposure [46, 56]. In contrast, findings from high‐income regions are mixed, which may be attributed to better nutrition, fortification programs, and a lower disease burden.

Nevertheless, these results must be regarded with caution. Confounding factors—including other nutritional deficiencies, geographical location, seasonal changes, ongoing health issues, and socioeconomic inequalities—frequently hinder causal interpretations. Additionally, reverse causation cannot be overlooked, as infections and inflammation can temporarily decrease serum 25(OH)D levels [57]. Emerging multi‐omics studies suggest that interactions between vitamin D, host genetics, epigenetic marks, and the respiratory microbiome can predict susceptibility to pneumonia, explaining the differences in results across various studies [52, 58]. Despite these drawbacks, the alignment of global data highlights vitamin D deficiency as a modifiable risk factor in the development and advancement of pediatric pneumonia.

## 5 Vitamin D Supplementation in the Prevention and Treatment of Pediatric Pneumonia

Vitamin D supplementation has been widely recommended to prevent deficiency and support normal immune function in children [59, 60]. Public health guidelines generally suggest routine supplementation to keep serum 25(OH)D levels adequate. This is especially important for groups at risk due to limited sunlight exposure, poor dietary intake, or maternal deficiency during pregnancy [30, 61]. It is crucial to differentiate between routine supplementation aimed at preventing vitamin D deficiency and therapeutic doses used during acute illness.

### 5.1 Preventive Supplementation

Clinical studies examining the preventive impact of vitamin D supplementation on respiratory infections in children have shown mixed and, in some cases, promising results [46, 48]. Several randomized controlled trials (RCTs) from low‐ and middle‐income countries (LMICs), such as Afghanistan and India, found that regular vitamin D supplementation slightly reduced the risk of recurrent pneumonia and acute lower respiratory infections [46, 48]. However, studies in high‐income countries, where vitamin D deficiency is less common, typically show limited preventive benefits [62, 63].

Meta‐analyses indicate that vitamin D supplementation might offer slight protection against acute respiratory infections. This effect seems stronger in individuals with severe vitamin D deficiency and those taking regular daily or weekly doses rather than large intermittent doses [27, 52]. Seasonal changes can also affect outcomes, with supplementation during the winter months likely providing better protection due to less sunlight [64].

### 5.2 Therapeutic Supplementation during Pneumonia

When it comes to therapeutic use, the results of vitamin D supplementation during acute pneumonia have varied. Some studies show that high doses of vitamin D given to deficient children can shorten recovery time, lower inflammatory markers, and reduce hospital readmissions [65, 66]. Other trials, such as [46] and [67], however, reported no significant improvement in clinical outcomes, including illness duration or mortality rates. Differences in initial vitamin D levels, dosing patterns, age groups, and coexisting malnutrition may help explain these mixed results.

It is important to distinguish between therapeutic vitamin D use during acute infection and standard public health supplementation strategies. Therapeutic protocols often include short‐term, higher doses under clinical supervision and should not be seen as general supplementation advice [27].

Current public health guidelines stress the importance of maintaining adequate vitamin D levels as part of broader child health strategies. The World Health Organization (WHO) and pediatric associations recommend keeping serum 25(OH)D levels at least 50 nmol/L, usually through supplementation of about 400–600 IU/day in children. This is particularly critical in areas with low sunlight exposure or a high incidence of infectious diseases [68, 69, 70]. However, the best dosing strategy during acute pneumonia is still unclear. This underscores the need for standardized, age‐specific randomized controlled trials to better understand the preventive and therapeutic roles of vitamin D supplementation in children.

## 6 Emerging and Mechanistic Insights

Recent studies have revealed additional factors through which vitamin D levels, genetic predispositions, microbiome interactions, and the synergy of micronutrients may affect the risk of pneumonia and immune responses in children (Table 3). Epigenetic regulation of immune genes affected by vitamin D exposure may explain why people have different risks and severities of infections [79, 80]. DNA methylation in VDR promoter regions or modifications to histones can adjust cytokine responses, antimicrobial peptides (AMPs), and barrier functions [79, 80].

**Table 3:** Emerging mechanistic insights on vitamin D and pediatric pneumonia.

Mechanistic domain	Biological focus/Key findings	Reference
Maternal and neonatal status	Low maternal 25(OH)D during pregnancy linked with increased infant ALRI and pneumonia risk; neonatal deficiency associated with severe bronchiolitis.	[71, 72]
Genetic factors (VDR polymorphisms)	VDR *FokI, TaqI*, and *BsmI* variants linked with pneumonia severity and immune response modulation; interaction with vitamin D deficiency worsens outcomes.	[73, 74]
Vitamin D–microbiome Interaction	Vitamin D supplementation delays pneumococcal carriage and alters airway microbial density; VDR signaling maintains epithelial–microbial homeostasis.	[40, 75]
Epithelial and barrier regulation	Vitamin D enhances tight junction integrity and reduces pathogen translocation in airway epithelia; mitigates RSV‐ and influenza‐induced damage.	[37, 38]
Synergistic micronutrient effects	Co‐deficiencies of vitamin D, zinc, and vitamin A elevate ALRI risk; combined supplementation lowers infection incidence.	[76, 77]
Epigenetic and molecular modulation	Vitamin D regulates expression of innate immune genes (e.g., *CAMP, DEFB4*) via VDR/RXR pathways and histone acetylation in airway cells.	[43, 78]

Studies involving cohorts demonstrate that insufficient maternal vitamin D during pregnancy correlates with an elevated risk of ALRI in infants. For example, a recent study conducted in South Asia indicated that infants of mothers with low vitamin D levels experienced approximately three times greater incidence of ALRI compared to those with adequate maternal vitamin D [71]. Another investigation found that neonatal levels of 25(OH)D below 50 nmol/L are linked to heightened severity of bronchiolitis and LRTIs [72].

Genetic variations within the vitamin D receptor (VDR) gene have been associated with an increased risk or severity of pulmonary diseases in children. A case‐control study from Egypt identified that the C‐variant of the FokI polymorphism in the VDR gene, combined with vitamin D deficiency, correlated with higher complication rates in cases of community‐acquired pneumonia [73]. Additional research also showed a relationship between VDR polymorphisms and the severity of wheezing or asthma attacks in children, suggesting similar pathways of immune dysregulation relevant to pneumonia [74].

Research into the interplay between vitamin D and the microbiome has explored maternal vitamin D supplementation effects on pneumococcal carriage in infants in Bangladesh [40]. The findings suggest that vitamin D intake can delay pneumococcal acquisition and alter carriage density in a dose‐dependent manner, indicating that vitamin D may affect airway microbial composition and subsequently reduce the early risk of invasive pneumococcal disease [40]. Reviews investigating vitamin D/VDR signaling underscore that vitamin D plays a role in preserving epithelial barriers and mucosal defenses, which, in turn, impacts the composition of both respiratory and gut microbiota [75]. Recent studies using microbiome sequencing show that vitamin D status affects not only pneumococcal colonization but also overall microbial diversity and resilience, highlighting its wider role in airway health [81, 82].

Children at higher risk for pneumonia often experience multiple nutrient deficiencies. A study performed in Bangladesh found correlations between vitamin D, vitamin A (retinol), and zinc levels with the risk of ALRI in underweight children aged 6 to 24 months [76]. Additionally, trials involving simultaneous supplementation of zinc and vitamin A in preschool children showed declines in the incidence of upper respiratory infections, suggesting potential additive or synergistic effects on immune function and barrier integrity [77]. Overall, the above study findings highlight that intervention strategies may need to take a comprehensive approach—considering maternal vitamin D status, genetic vulnerabilities, microbiome influences, and nutritional synergies—to enhance the prevention and management of pediatric pneumonia.

## 7 Gaps and Future Directions

Despite accumulating evidence, there are still significant gaps that hinder its translation into clinical settings.

Standardization of measurement and thresholds: Currently, there is no established global agreement on the serum 25(OH)D levels indicating deficiency, insufficiency, and sufficiency in pediatric populations. A recent meta‐analysis focused on defining thresholds for rickets found that minimal risk levels were around 28 nmol/L for children with sufficient calcium intake and 40 nmol/L in cases of inadequate calcium intake [83]. Assay variability among laboratories adds further complexity, as seen in Germany's health surveys, which demonstrated that standardizing the measurement of 25(OH)D led to significant changes in the estimated rates of deficiency [84].

Longitudinal cohorts and multicenter RCTs: There is a need for large, prospective cohort studies that observe children from birth through early childhood across various geographic locations to gather data on incidence, severity, and long‐term respiratory health outcomes. An example of this is the “d‐Kids” study protocol targeting First Nations populations, which aims to decrease acute respiratory infections through perinatal vitamin D supplementation [85]. Large‐scale multicenter RCTs should provide clarity on the optimal dosage, timing (before vs after infection), and the identification of high‐risk groups.

Genetic and epigenetic modifiers: To date, there has been limited investigation into how genetic variations in VDR or vitamin D binding protein (DBP), as well as epigenetic factors, or the interactions with the host microbiome, influence the effect of vitamin D on respiratory infections. These aspects represent promising opportunities for precision approaches.

Future research should combine developmental immunology, epigenetics, and microbiome dynamics to improve age‐specific supplementation strategies. Multicenter studies should consider seasonal and environmental factors, host genetic differences, and co‐nutrient status.

Implementation studies in LMICs should evaluate combined approaches. These include maternal supplementation, early‐life infant dosing, and environmental changes to improve vitamin D effectiveness and lower the rate of pediatric pneumonia.

Integration into policy and child health programs: Although there are existing guidelines in some countries for the prevention of vitamin D deficiency and treatment of rickets, they have not yet formally incorporated pneumonia or acute respiratory infections as key outcomes. Public health initiatives should integrate vitamin D supplementation into child health strategies, particularly in low‐ and middle‐income countries, along with measures for nutrition, sun exposure, and infection control.

## 8 Conclusions

Vitamin D plays multiple roles in pediatric pneumonia. It is both an essential nutrient and a key player in the immune and epithelial defenses of the respiratory tract. Through signaling via vitamin D receptors, it boosts antimicrobial peptide production, adjusts inflammatory cytokines, and keeps the airway epithelial barrier strong. This helps protect against respiratory pathogens. Evidence from epidemiological studies and clinical trials shows that a lack of vitamin D is linked to a higher risk and more severe cases of pneumonia in children, especially in groups where deficiency is common.

The effect of vitamin D seems to differ at various developmental stages. These differences relates to factors such as immune development, hormonal balance, and environmental influences. Emerging evidence also points to the importance of a mother's vitamin D levels, genetic differences in vitamin D signaling, early immune development, and connections with the respiratory microbiome. These factors may help explain the variations seen in clinical outcomes and intervention studies. Improving vitamin D status could serve as a valuable additional strategy for children's respiratory health. Future studies should focus on long‐term cohorts, age‐specific supplementation trials, and combined multi‐omics approaches. This will help clarify the mechanisms involved and lead to better prevention strategies for childhood pneumonia.

## Author Contributions

**Nurshad Ali:** conceptualization, writing – review and editing, writing – original draft.

## Ethics Statement

This article is a review of existing literature and does not contain any original data involving human or animal participants.

## Conflicts of Interest

The author declares no conflicts of interest.

## Transparency Statement

The lead author Nurshad Ali affirms that this manuscript is an honest, accurate, and transparent account of the study being reported; that no important aspects of the study have been omitted; and that any discrepancies from the study as planned (and, if relevant, registered) have been explained.

## Data Availability

Data sharing not applicable to this article as no datasets were generated or analysed during the current study.
